# Model predictive control of DC/DC boost converter with reinforcement learning

**DOI:** 10.1016/j.heliyon.2022.e11416

**Published:** 2022-11-05

**Authors:** Anup Marahatta, Yaju Rajbhandari, Ashish Shrestha, Sudip Phuyal, Anup Thapa, Petr Korba

**Affiliations:** a Department of Electrical and Electronics Engineering, Kathmandu University, Dhulikhel 45200, Nepal; b Department of Electrical Engineering, Information Technology and Cybernetics, University of South-Eastern Norway, Porsgrunn N-3918, Norway; c School of Engineering, Zurich University of Applied Science, DH-8401 Winterthur, Switzerland

**Keywords:** Power electronic converters, Non-linear controller, Microgrid, Machine learning

## Abstract

Power electronics is seeing an increase in the use of sophisticated self-learning controllers as single board computers and microcontrollers progress faster. Traditional controllers, such as PI controllers, suffer from transient instability difficulties. The duty cycle and output voltage of a DC/DC converter are not linear. Due to this non-linearity, the PI controller generates variable levels of voltage fluctuations depending on the operating region of the converter. In some cases, non-linear controllers outperform PI controllers. The non-linear model of a non-linear controller is determined by data availability. So, a self-calibrating controller that collects data and optimizes itself as the operation goes on is necessary. Iteration and oscillation can be minimized with a well-trained reinforcement learning model utilizing a non-linear policy. A boost converter's output power supply capacity changes with a change in load, due to which the maximum duty cycle limit of a converter also changes. A support vector calibrated by reinforcement learning can dynamically change the duty cycle limit of a converter under variable load. This research highlights how reinforcement learning-based non-linear controllers can improve control and efficiency over standard controllers. The proposed concept is based on a microgrid system. Simulation and experimental analysis have been conducted on how reinforcement learning-based controller works for DC-DC boost converter.

## 1 Introduction

In recent years, the adoption of renewable energy resources and power electronic technologies have been increased massively because of the concept of clean energy and the flexibility of the power electronic technologies, which also increase the viability of the DC microgrid system [1, 2]. A DC microgrid consists of distributed energy resources that contribute power to the grid. A microgrid faces many problems related to power quality and system dynamics and becomes unstable mainly due to load fluctuations and uncertain power generation which lead to bus voltage fluctuation [3, 4, 5]. The DC/DC converters are the backbone of a DC microgrid, since the power generators are connected to the grid via DC/DC converters. In the AC grids, DC-AC inverters are used to connect the power generators to the grid. However, due to variable levels of voltage generated by these power generators, the DC/DC converters have to be used to provide constant input voltage for the inverter [6], which makes DC/DC converters a vital part of renewable energy sources-based power systems. The problem of regulating the output voltage of these converters has been of great interest for many years. These converters can be categorized mainly into three categories: buck converters, boost converters, and buck boost converters [6, 7, 8, 9]. Uncertain power generation, consumption, and non-linearity of the system make it challenging for the controllers and converters to maintain constant voltage in conditions in the normal operation of the system as well as in contingencies [10, 11]. In order to achieve the proper voltage regulation in a DC microgrid, the controllers like PID controllers, model predictive controllers, sliding mode controllers, fuzzy logic-based controllers, and neural network-based controllers can be used [3, 12].

In a DC/DC boost converter, the semiconductor switches are the most important components; the output of a boost converter is controlled by controlling the duty cycle of the switching pulse supplied to the semiconductor switch, and the duty cycle of the supplied pulse is controlled by a controller [4]. The main task of the controller is to maintain the stable transient and steady-state response in the output of the converter by controlling the pulse width and frequency of control signals fed to the semiconductor switch [13, 14]. The PID controllers are the most common type of controller used for voltage regulation of DC/DC converters, which are most popular due to their compatibility and flexibility to implement specific characteristics of energy systems [6]. PI controllers can have a fast response time and a good steady-state response, but transient voltage stability is compromised with response time. A dynamic and complex system that requires high stability has demanded these types of controllers, hence, there is a need for a robust controller which can adapt itself to control specific systems [13, 15].

Over the few past decades, several new control systems like sliding mode controllers, fuzzy logic controllers, neural network-based controllers, fuzzy neural controllers, and deep reinforcement learning-based controllers have been introduced. The most highlighted feature of the sliding mode controller is its inherent variable structure and the most negative point is the variable switching frequency [6, 16]. Fuzzy logic-based controllers lack formal analysis and are not considered reliable controllers, hence, the adaptive fuzzy and model predictive controllers have been studied as a replacement for the fuzzy logic controller. The model predictive controller is a suitable controller for nonlinear systems, but its performance is highly dependent on the system model [13], which means that the system has to be manually modeled before implementing the controller. On the other side, neural network-based controllers are highly dependent on training data provided to them [17]. Depending upon the area of application the input supply characteristics (like input voltage range and power supply capacity) and output load characteristics may vary. In these conditions the conventional controllers need to be re-calibrated for optimal performance [3, 15] Using reinforcement learning enables the controller to self-model the system it is being applied to and use the system model to control it without outside intervention which makes the system more robust. Reinforcement learning based controllers are more versatile than traditional controllers due to their self-calibration capabilities [12]. The deep reinforcement learning model interacts with the environment and tries to develop the best policy; depending on what action a controller took and what response it got from the environment, the controller gets the data about the system it is controlling [3, 4]. This data and the neural network are used by the controller to develop a policy function. However, the neural network is a complex system that uses large computing power and can be complex to replicate the policy on other controllers with similar specifications. An alternative to this can be a regression-based model to determine the optimal policy, which can generate policies in the form of simple formulas with a lesser number of variables [3, 18, 19, 20]. Because of this, controllers can be easily replicated compared to neural networks-based functions.

This paper presents a robust control method to control the DC/DC boost converter output. A reinforcement learning-based controller, utilizing a non-linear predictive model as a policy has been proposed in this paper.

Unlike conventional reinforcement learning models utilizing deep neural networks, this work purposes a simpler regression-based optimization method that requires comparatively low computing power and can even be implemented in a microcontroller for DC-DC converters' control purposes. This study also proposes reinforcement learning based fault detection system for the DC-DC boost converter for efficient operation of the converter. The controller uses non-linear regression to optimize the policy function. Simulation and experimental analysis of a proposed controller have been conducted to verify the performance. The response of the proposed controller has been compared with the response of traditional controllers to verify the results.

The proposed controller can also be applied to other power electronics converters like buck and buck-converters to provide better stability. Though described in detail following are the main contributions of this paper:

a. A robust non-linear controller based on reinforcement learning that uses a regression-based optimization algorithm has been proposed to reduce the transient oscillation and settling time of the DC/DC boost converter during load fluctuations. After conducting the results, the performance of the controller is found to be improved over the standard controller.
b. A hybrid model has been proposed that combines a non-linear model and an integral controller to improve the transient and steady-state stability in comparison to the classic PI controller.
c. The proposed model has been tested under both simulation and laboratory-based environments. A hardware prototype has been developed in the laboratory to check the performance of the model, and compare the results with the simulated results. The validity of the proposed controller is verified in both simulation and hardware.

The overall structure of this paper is organized as follows: Section 1 presents the general overview of the DC/DC converters with their issues and potential improvements. Section 2 gives the theoretical backgrounds of the technologies. The adopted methodology is described in Section 3. The simulation and experimental results are discussed in Section 4. The conclusion of this study is presented in Section 5.

## 2 Theoretical background

### 2.1 DC/DC boost converter

**Figure 1:**
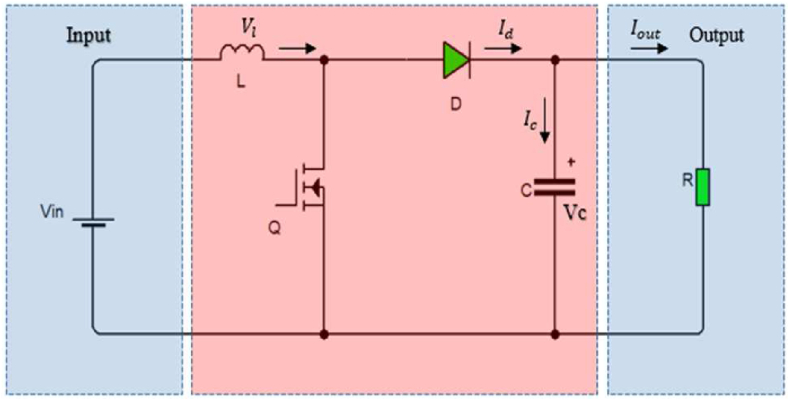
Boost converter circuit.

A general circuit of a boost converter is shown in Figure 1. The boost converter consists of a high-frequency power switch that charges and discharges the inductor L and capacitor C, through two power electronics switches: a controllable switch Q and a diode D. In this model, the diode on-time resistance, the equivalent series resistance of the capacitor, and switch on-time resistance are ignored. The output voltage of the converter is controlled by controlling the duty cycle of high-frequency input pulses; higher the frequency of the PWM pulses lowers will be the size of the inductor required. The maximum and minimum duty cycle that is required by a boost converter is given by Eq. (1). The power electronic switch quickly charges the inductor to high voltages, and then the inductor will in turn charge the capacitor. The inductor can charge the capacitor to the required voltage level within the ripple voltage limit, as long as the load connected to the output draws the current in such a way that the required output voltage level draws lesser output power than the input power supplied. Theoretically, input power supplied should be equal to output power drawn, but due to switching and magnetic losses output power is always lesser than the input power.

(1) $$D_{\max}=1-\frac{{Vin}_{\min}*\eta }{Vout}$$

The minimum inductor size required to achieve the required output voltage *Vout* for an input voltage of *Vin* is given by Eq. (2), and the inductor ripple current *ΔI*_*l*_ is given by Eq. (3) [4].

(2) $$L=\frac{Vin*\left(Vout-Vin\right)}{{\Delta I}_{1}*f*Vout}$$

(3) $${\Delta I}_{1}=\left(0.2\;to\;0.4\right)*{Iout}_{\max}*\frac{Vout}{Vin}$$

In Eq. (2), *ΔI*_*l*_ is the inductor ripple current and *f* is the switching frequency of the converter. The minimum size of the output capacitor required in order to achieve the ripple voltage of *ΔVout* is given by Eq. (4) [4].

(4) $${Cout}_{\min}=\frac{{Iout}_{\max}*D}{f*\Delta Vout}$$

A boost converter has a non-linear relation between the duty cycle and output voltage due to the non-linear characteristics of the inductor. When an inductor changes, the voltage drops across it decreased exponentially and the current through it increases exponentially. The inductor current and voltage have an exponential relation with charging time. The exponential charging characteristics of a boost converter can be seen in Eqs. (5) and (6). In a boost converter, the output capacitor charging voltage can be given by Eq. (7) [4].

(5) $$V_{l}=V_{in}*\;e^{-\frac{Rt}{L}}$$

(6) $$I_{l}=\frac{V_{in}}{R}*\left(\left(1-e^{\frac{-Rt}{L}}\right)\right)$$

(7) $$Capacitor\;charging\;voltage=V_{in}+V_{l}$$

It can be seen from Eqs. (5) and (6) that the charging time of the inductor is exponentially related to the inductor voltage ${\begin{array}{c} V \end{array}}_{l}$ and inductor current $I_{l}$. The output voltage of a boost converter is directly proportional to the voltage dropped across the inductor. For a fixed operating frequency, the duty cycle and output voltage are also going to be related exponentially. This also creates non-linearity between the output voltage and PWM duty cycle in a boost converter.

**Figure 2:**
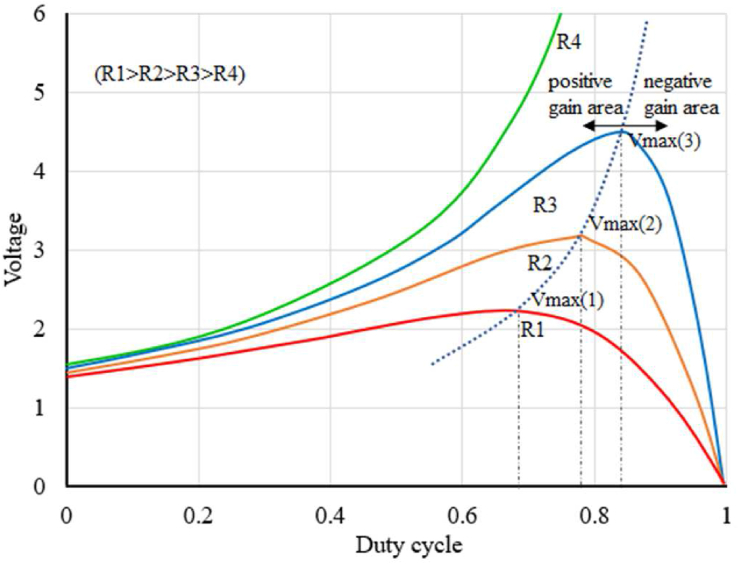
Boost converter duty cycle vs output voltage for different loads.

When the duty cycle is increased for a boost converter the output voltage increases up to a certain point and beyond that point, it starts to decrease. This duty cycle vs voltage curve varies according to the load connected to the converter [21, 22]. In order to run the converter with maximum efficiency, it should be run on the left side of the peak voltage region. Figure 2 shows how the duty cycle affects the voltage output of a converter for different loads. As it can be seen from Figure 2, the operating region of a boost converter can be separated into two regions, positive and negative gain regions [21]. A converter should be run in a positive gain region for efficient operation. For variable loads positive and negative gain regions can be separated by a support vector. In this experiment, a non-linear regression is used to determine this support-vector. This vector can be used to determine whether the converter should be operated in the given region [22, 23].

### 2.2 Reinforcement learning

Reinforcement learning is a self-learning intelligent method in which an agent interacts with the environment to map certain state spaces to corresponding action spaces. For a completely observable environment, reinforcement learning can be described by the Markov decision process under the Markovian characteristic of the environment. The Marconian decision-making process is described by five tuples (*S, A, P, R, Y*), where *S* is the state space, *A* is the action space, *P:S∗A∗S* is state transition provability, *R:S∗A* is the reward function, and *Y* is the discount function. The target of interacting with the environment is to maximize the cumulative reward over the period of training [3, 15]. When the destination can be reached with a single step from every state, the optimal expected action-value function can be determined by Eq. (8). Here, γ is the discount factor and $R_{t_{k+1}}$ is the reward given for reaching the state $S^{\prime }$ from *S* by taking action *A* [3].

(8) $$G_{t}=\sum_{k=0}^{n}\gamma^{k}*R_{t_{k+1}}$$

The main goal of a reinforcement learning model is to map the correct state-action pairs. To do this, an optimal action-value matrix also called *Q* matrix is created and the value of this matrix is updated while the algorithm expl

ores the environment. The optimal expected action-value function of a state *S* and action *A* is given by the bellman optimality Equation as given by Eq. (9) [3]. Here (*S’, A’*) are the next possible state-action pair that will give the maximum state action value for state *S’*; if policy *π* is followed while updating *Q* matrix, Eq. (9) is used to update the value of *Q (S, A).* Eq. (9) only takes the future and present rewards into consideration but not the past, hence, there is a need for a factor that tells how many past rewards to keep and how many future rewards should affect the current decision. The learning rate *α* is introduced to provide information on the factor. Similarly, to limit the effect of future rewards, the discount rate γ is used. After considering these parameters, the action-value function is given by Eq. (10) [3].

(9) $$Q_{*}\left(S,A\right)=E\left[\;R_{t+1}+\gamma *\;\max_{\pi }Q\left(S^{\prime }+A^{\prime }\right)\right]$$

(10) $$Q_{*}\left(S,A\right)=\left(1-\alpha \right)+\alpha \left(R_{t+1}+\gamma *\;\max_{\pi }Q\left(S^{\prime }+A^{\prime }\right)\right)$$

**Figure 3:**
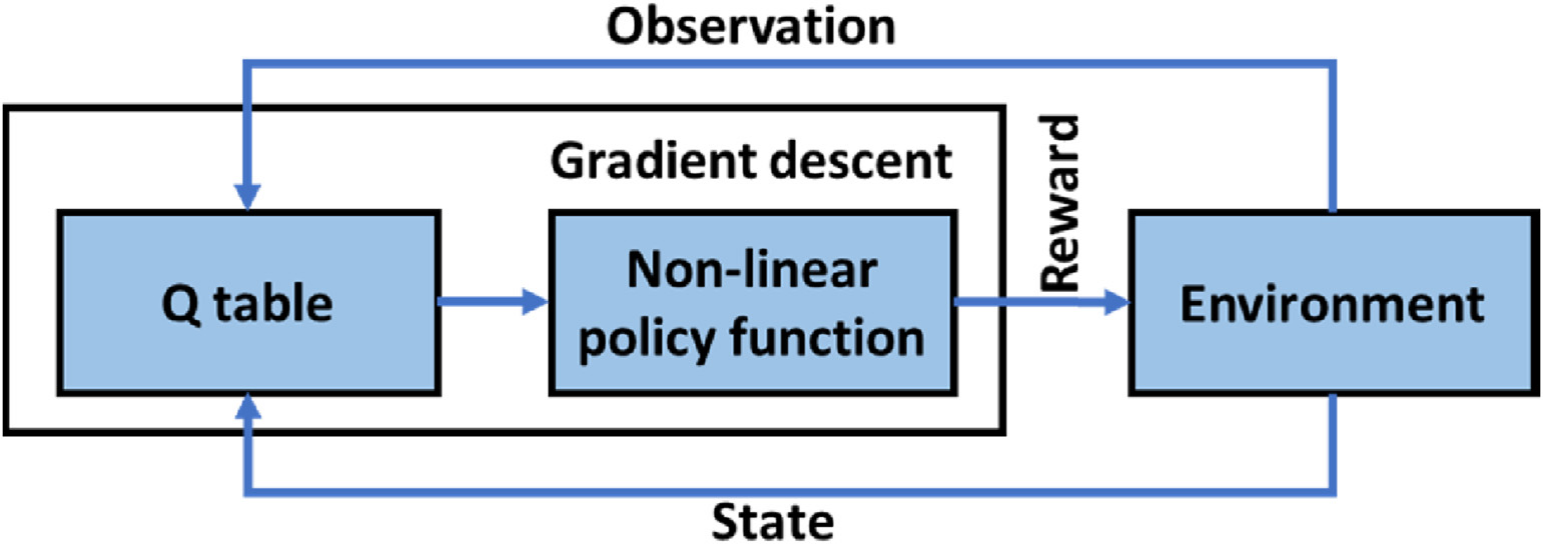
Reinforcement learning model.

In a reinforcement learning model, an agent interacts with the environment via action and gets a reward for that specific state-action pair. To take suitable action for an encountered state, the reinforcement learning model should have interacted and trained itself with the environment for that specific state. For an environment where infinite states are possible, the model cannot work, hence, there is a need for a policy. In this paper, the authors use non-linear regression and optimal values from the *Q* table to determine the suitable policy. The logic behind the reinforcement learning model is given in Figure 3.

A boost converter has nonlinear relation between the duty cycle and output voltage for a given load as shown in Figure 2. A PI controller will give various levels of voltage fluctuation depending upon its operating region. It will perform optimally only for the particular region where the controller is calibrated. While considering a nonlinear model-based controller will give optimal performance for every operating region and using reinforcement learning to model the non-linearity between duty cycle and output voltage for a given load will eliminate the need to calibrate the controller by the user, which would not be possible using the conventional controllers.

### 2.3 Regression

Regression can be used in power electronics converters to identify a relationship between input and output variables, and the main goal of implementing regression in power electronics converters is to predict the value of the input signal to achieve the target output [24]. Depending upon the nature of converters, the relation between input and output variables varies. Some relations might be solved with linear regression, while some may require non-linear regression. Linear regression requires fewer steps and lower computing power to solve than non-linear regression. When all terms in a model are either a constant or a parameter multiplied by an independent variable, the regression model is supposed to be linear. When the relation can be mapped by a polynomial or simple exponential function, the linear regression method is the best way to solve it. A polynomial with degree *n* can be represented by Eq. (11) [19]. These data can be represented in matrix-vector form as given in Eq. (12) [18, 19].

(11) $$Y=C1x^{n}+C2x^{n-1}+C3x^{n-2}+\ldots \ldots \ldots \ldots ..\ldots \ldots \ldots ..+Cn$$

(12) $$\left[\begin{array}{cc} \begin{array}{cc} {x1}^{n} & {x1}^{n-1}\\ {x2}^{n} & {x2}^{n-1} \end{array} & \begin{array}{cc} {x1}^{n-2} & \begin{array}{cc} \ldots & 1 \end{array}\\ {x2}^{n-3} & \begin{array}{cc} \cdots & 1 \end{array} \end{array}\\ \begin{array}{cc} \vdots & \vdots \\ {xm}^{n} & {xm}^{n-1} \end{array} & \begin{array}{cc} \vdots & \begin{array}{cc} \ddots & \vdots \end{array}\\ {xm}^{n-2} & \begin{array}{cc} \cdots & 1 \end{array} \end{array} \end{array}\right]\left[\begin{array}{c} C1\\ \begin{array}{c} C2\\ \begin{array}{c} \vdots \\ Cm \end{array} \end{array} \end{array}\right]=\left[\begin{array}{c} Y_{1}\\ \begin{array}{c} Y_{2}\\ \begin{array}{c} \vdots \\ Y_{M} \end{array} \end{array} \end{array}\right]$$

Here Eq. (12) can be represented as [A][C]=[Y], where the values of *C* can be calculated through Eq. (13) [25].

(13) $$\left[C\right]=\left[Y\right]\;{\left[A\right]}^{T}\left[\left[A\right]\;{\left[A\right]}^{T}\right]$$

If a regression model does not fulfill the criteria of a linear regression model then it has to be linearized first and then solved using a non-linear regression model. While solving for functions that cannot be represented directly in the linearized form, the function has to be linearized first and then solved. An example of a function that needs a non-linear regression method to be solved is shown by Eq. (14) [18].

(14) $$y=f\left(x,C_{1},C_{2}\right)=C_{1}e^{bC_{2}}$$

For *m* number of data, Eq. (14) can be re-written as Eq. (15). Where *R* is the difference between the actual value and the predicted value. After partial differentiation of Eq. (15) with respect to $C_{1}\;and\;C_{2}\text{,}$ this relation can be written as Eq. (16) and can be solved via Eq. (17) [20, 25, 26].

(15) $$R_{i\;}=y_{i}-f\left(x_{i},C_{1},C_{2}\right)$$

(16) $$\begin{array}{cc} x_{1} & \to \\ x_{2} & \to \\ \begin{array}{c} \vdots \\ x_{m} \end{array} & \begin{array}{c} \to \\ \to \end{array} \end{array}\left[\begin{array}{c} \begin{array}{cc} \frac{df}{dC_{1}} & \frac{df}{dC_{1}} \end{array}\\ \begin{array}{cc} \frac{df}{dC_{1}} & \frac{df}{dC_{1}} \end{array}\\ \begin{array}{c} \begin{array}{cc} \vdots & \vdots \end{array}\\ \begin{array}{cc} \frac{df}{dC_{1}} & \frac{df}{dC_{1}} \end{array} \end{array} \end{array}\right]\left[\begin{array}{c} \Delta C_{1}\\ \begin{array}{c} \Delta C_{2}\\ \begin{array}{c} \vdots \\ \Delta C_{m} \end{array} \end{array} \end{array}\right]=\left[\begin{array}{c} Y_{1}\\ \begin{array}{c} Y_{2}\\ \begin{array}{c} \vdots \\ Y_{M} \end{array} \end{array} \end{array}\right]$$

Here Eq. (16) can be represented as [A][C]=[Y]*,* and can be solved by the matrix-vector multiplication method as given in Eq. (17).

(17) $$\left[\Delta C\right]=\left[Y\right]A^{T}\left[\left[A\right]{\left[A\right]}^{T}\right]$$

While deep reinforcement-based controllers perform well in computers with good processing powers, the same method cannot be applied to end devices like microcontrollers due to their limited computing power. So, a simpler Q-table-based method along with a regression-based optimization technique, which can easily be applied with the low computing power of a microcontroller has been used in this study.

## 3 Method

This paper proposes a reinforcement learning-based control system that uses regression to determine the best policy for the controller. In this study, the reinforcement learning model has been used to map the boost converter duty cycle and load impedance connected to the converter and to determine the support vector separating the positive gain region and the negative gain region as shown in Figure 2. The relation between duty cycle and load impedance is not linear. Hence, the model uses a second-order exponential equation as the policy to generate the required PWM signal. Similarly, a third-order non-linear equation is used as a policy function to separate positive gain and negative gain regions. The system uses nonlinear regression-based optimization to optimize the policy function. The reinforcement learning model optimizes the policy by using the data from the *Q* table with the help of non-linear regression as discussed in section 2. The model uses the impedance of the output load as the state and the duty cycle of the PWM signal as the action. The model consists of an instantaneous load impedance tracking loop and a voltage tracking loop. In order to track the load impedance, shunt voltage drop and overall output voltage of the converter are used. The instantaneous load impedance tracking loop measures the load connected to the converter by using the shunt load voltage drop and overall output voltage. Initially, the controller will not be able to generate PWM signal as required due to a lack of data in the *Q* table, so an integral controller-based compensator is used to compensate for the error generated by the reinforcement model. As the model trains itself over the large-no-states, the policy will be optimized and the response of the controller will become faster and the compensator will have less and less contribution to the PWM signal generated by the controller. However, the controller cannot be mapped perfectly due to an infinite number of potential states and other practical limitations. Hence, there will be some errors in the PWM signal generated by the policy function, which can be eliminated by the compensator.

**Figure 4:**
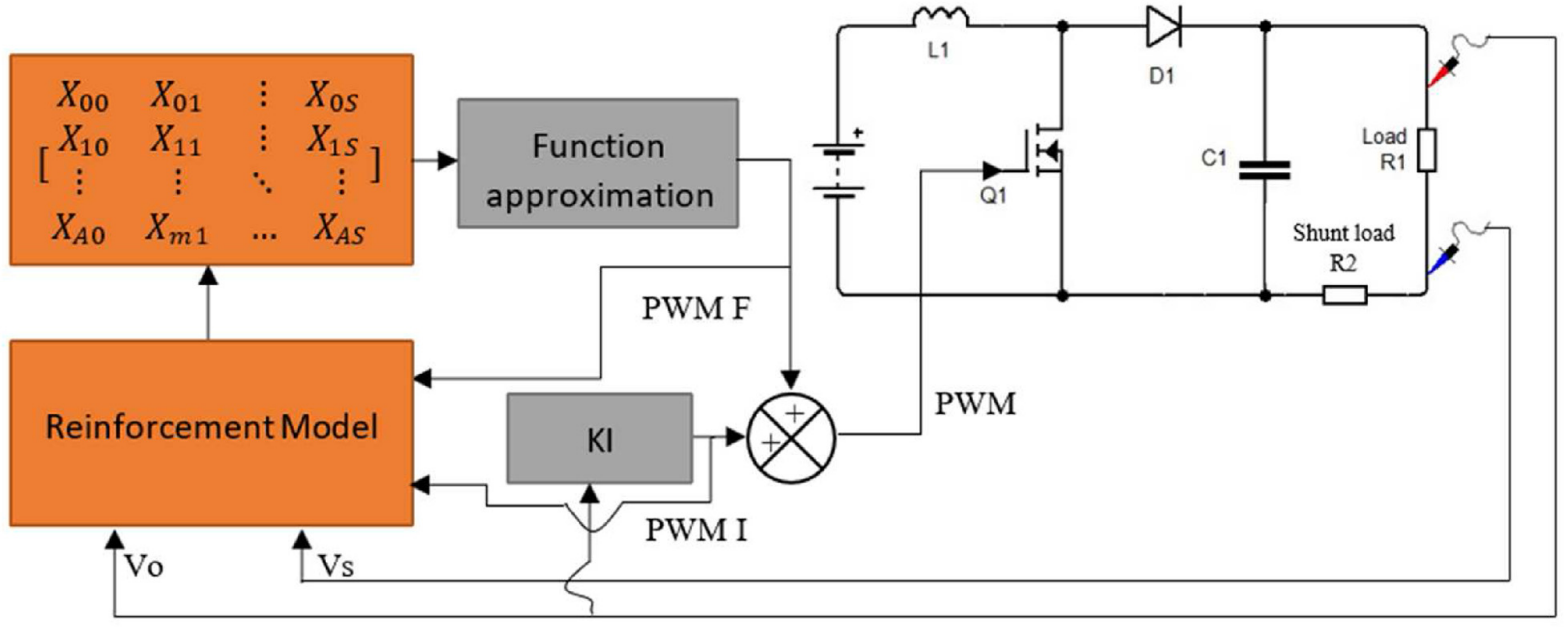
Block diagram of the control system.

**Figure 5:**
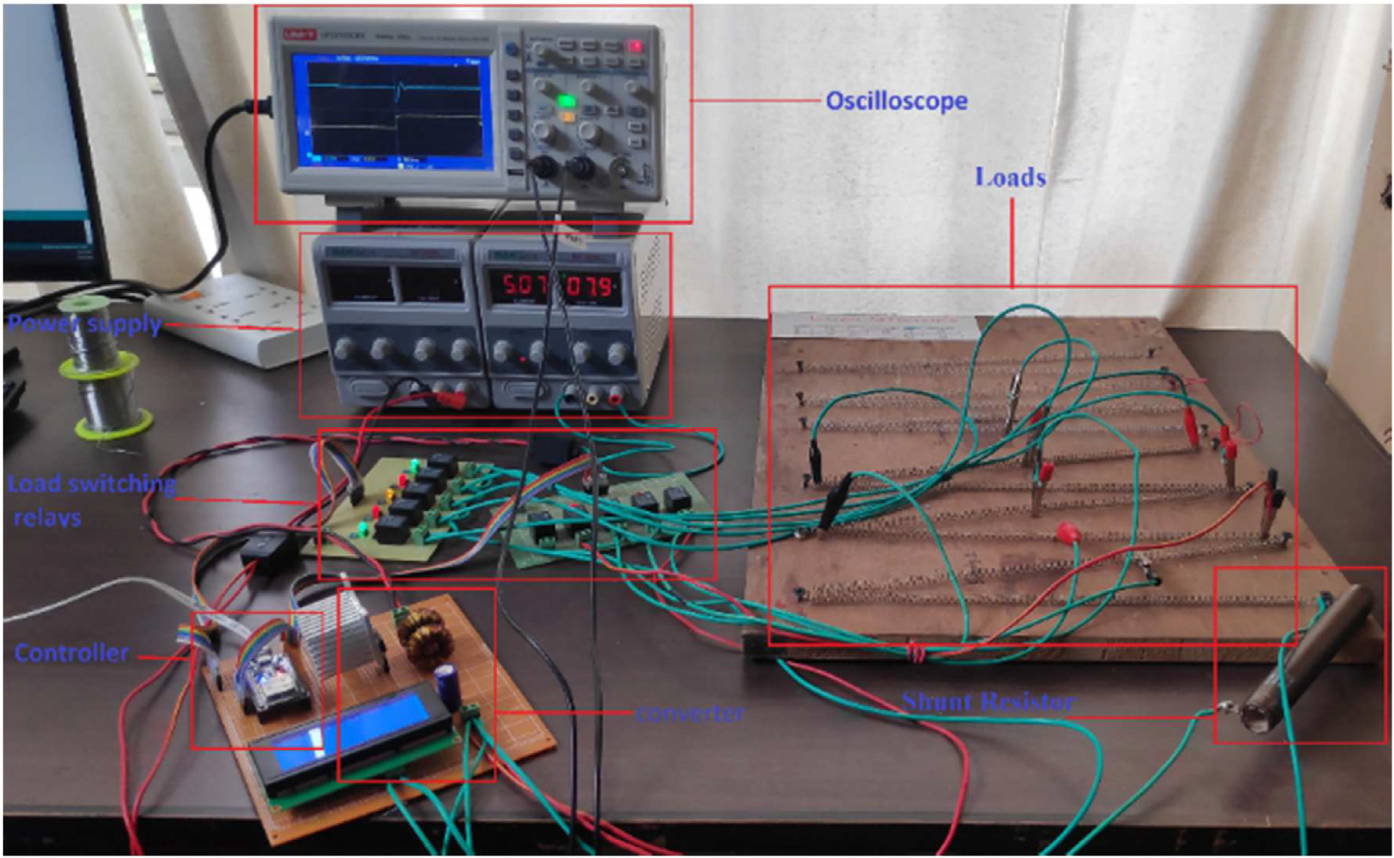
Experimental setup.

The controller uses the impedance of the load connected to the converter as the state and PWM as the action to the given state. The input-output relation is mapped between the output load impedance connected to the converter and the input PWM signal. Figure 4 shows how the controller is implemented in this paper. The reinforcement learning model interacts with the boost converter using the policy function. The converter calculates the load impedance by using Eq. (18). The experimental setup used is shown in Figure 5.

(18) $$R_{1}=\frac{V_{0}-V_{s}}{V_{s}}*R_{2}$$

Algorithm 1 shows how the proposed controller is implemented in this experiment. First, a reward matrix is defined which will be used to store rewards for respective state-action-pair. A tentative policy function is defined; the controller will try to optimize it. Then the program reads the data from the *Q* table and determines if the reward for a new state-action-pair is inserted: if yes then the program will iterate by using regression and try to optimize the policy function. The load impedance is taken and appropriate PWM is generated by the policy function which will be compensated by the I controller and written to the output of the microcontroller.

Algorithm 1 Operation of the control system

1 Start
2 define Q matrix
3 define a policy function
4 while true
5 read data from Q table
6 if n (S, A) > np (S, A)
7 for (i = 0; i < 50; i++)
8 Iterate for the best policy
9 np (S, A) = n (S, A)
10 read the load impedance
11 calculate PWM
12 write PWM
13 Update Q table
14 End

Algorithm 2 shows how the reinforcement learning model is implemented to update the *Q* table. The algorithm checks if the current action is better than the previous action for the given state: if true then it logs the reward for the current state-action-pair. For any state, the controller is capable of achieving the required output by taking only one action. Due to this, we do not need to consider the possible future reward and past rewards while rewarding any state-action pair.

Algorithm 2 Q table updating algorithm

1 Start
2 Read the voltage and PWM
3 if (voltage >23.6 && voltage <24.4)
4 Read maximum reward $\left(S_{Rmax}\right)$ for the current state
5 New reward $\left(S_{nR}\right)$ = |voltage – 24|
6 If $S_{Rmax}$ < $\left(S_{nR}\right)$
7 Update reward with $\left(S_{nR}\right)$
8 End

A PI controller generates the required PWM signal based on the magnitude of the error and accumulated total error during the run process. The duty cycle of the PWM signal generated by a PI controller can be shown in Eq. (19). The duty cycle of the PWM signal generated by the proposed controller is given by Eq. (20).

(19) $$D=\Delta V*K_{p}+K_{I}\sum_{0}^{t}\Delta V$$

(20) $$D=F\left(R_{L}\right)+K_{I}\sum_{0}^{t}\Delta V$$

In Eq. (19), $\Delta V{*K}_{p}$ calculates the approximate value of the required duty cycle for the given condition, and $K_{I}\sum_{0}^{t}\Delta V$ generates the required compensation signal to generate the required duty cycle. In a system consisting of a PI controller, the effect of the P and I controller on the control signal is continuous. A P controller generates linear PWM with respect to output voltage error and the integrator compensates. However, the relation between the errors in output voltage and the duty cycle is not linear. Because of this, the I controller behaves differently in the non-linear region than in the linear region. I controller generates higher overshoots in the non-linear region due to large accumulated errors. Eq. (20) uses a non-linear function approximated by the reinforcement model and compensated by the integral controller. Since the function is approximated by mapping the input and output of that specific device, theoretically function should be able to generate perfect output signals. However, due to imperfection in mapping and a finite number of state-action-pair used to approximate the function, the signal generated may not be perfect, hence, the integral controller is used to generate the compensation signals. The output signal generated cannot be perfect; an integral controller is paired with the model predictive controller to compensate for the steady-state errors. In Eq. (19), the integral controller has a varying effect on the outcome depending upon the connected load to the output. However, in Eq. (20), $F\left(R_{L}\right)$ is a non-linear function representing the relation between input and output, and the effect of the integral controller in output is very little and uniform over the varying load conditions. Hence, the overshoot and oscillation can be minimized by implementing the proposed controller. The proposed controller uses load impedance as feedback instead of voltage because load fluctuation is the cause of transient instability in a boost converter and voltage fluctuation is the response of the converter to the load fluctuation. Also, A boost converter consists of a capacitor in output to filter the high-frequency ac voltage. Due to this capacitive component, there is a lag in voltage change to the load change. The load impedance change can be measured accurately faster than output voltage change, hence, the actions can be taken faster and more accurately by implementing the proposed controller than the traditional controllers. In this model, the state of the system is taken as the load connected to the output of the converter, action is duty cycle fed to the gate of the MOSFET, and error is determined concerning output voltage generated by any state-action pair. The transfer function of the proposed controller is given by Eq. (21), where *R* is the load connected to the boost converter.

(21) $$D=ae^{bR_{1}}+{\;ce}^{DR_{1}}+K_{I}\sum_{0}^{t}\Delta V$$

Similarly, a reinforcement learning model is used to separate the positive gain region and the negative gain region of the converter. A third-order equation is used to separate the two regions. This equation is adjusted by the regression model as the converter is introduced to different load and duty cycle conditions. After the line separates the positive and negative gain regions, it determines the converter that will operate on positive gain area while avoiding the negative gain area which helps to run the converter with high efficiency. If the duty cycle is in the negative gain region, and the converter is maintaining the output voltage, then the converter is running with lower efficiency and supplies the same amount of power to the load with greater efficiency if operated in the positive gain region, if the converter is not maintaining required output voltage level then the converter shuts off. This feature can help to extend the life cycle of the converter as it prevents the converter from operating in the negative gain region. Further, the converter operates in the negative gain region, the less efficient it becomes and triggers the risk of damaging the MOSFET.

The benefit of using a regression model to determine the optimal policy function in comparison to a neural network is that the policy function generated is simpler than the neural network and it also requires less time to train because of its lower complexity. Once an optimal policy function is determined it is easier to replicate the controller for the converters with the same specifications.

## 4 Result and discussion

In order to determine the relationship between the duty cycle and the connected load, the reinforcement learning model is implemented and analyzed. To teach the model, the best policy data is required, for which the programmable load is used. The specifications used for the boost converter are shown in Table 1. First, simulation is performed in MATLAB-Simulink and texted in a hardware-based experiment. The detail of the conducted experiments is discussed in two sub-sections.

**Table 1:** Specifications of the converter.

Parameter	Value
Inductor	40 μH
Output capacitor	4000 μF
Switching frequency	10 kHz
Sampling frequency	1 kHz
Output voltage	24 V

### 4.1 Simulation outcomes

**Figure 6:**
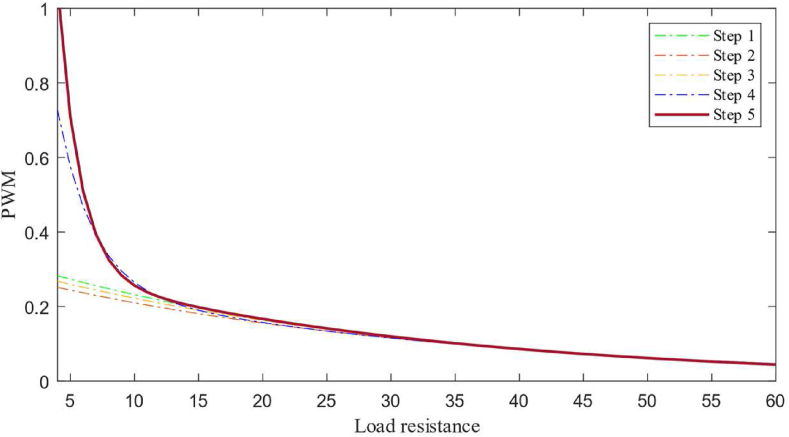
Policy developed at different stages (Simulation).

First, a random second-order exponential function is defined as the policy, and the policy function is paired with an integral compensator. The PWM data from the controller is fed to the boost converter. The loads connected to the output of the converter are programmed to switch in predefined time intervals. As the converter is connected to different levels of load, the policy function starts to optimize and the contribution of the compensator to the PWM signal starts to decrease. Figure 6 shows the policy function generated at the different levels of training stages.

Here in Figure 6, the steps (i.e., 1 to 5) represent the policy function at different stages of the training. In the future, if any new load condition arrives then the main policy function will change by a newly optimized policy. The policy function is generated by the controller in simulation as well as in hardware that maps the relation between load connected to the converter and duty cycle as given by Eq. (22).

(22) $$F\left(R_{L}\right)=ae^{bR_{L}}+ce^{dR_{L}}$$

**Figure 7:**
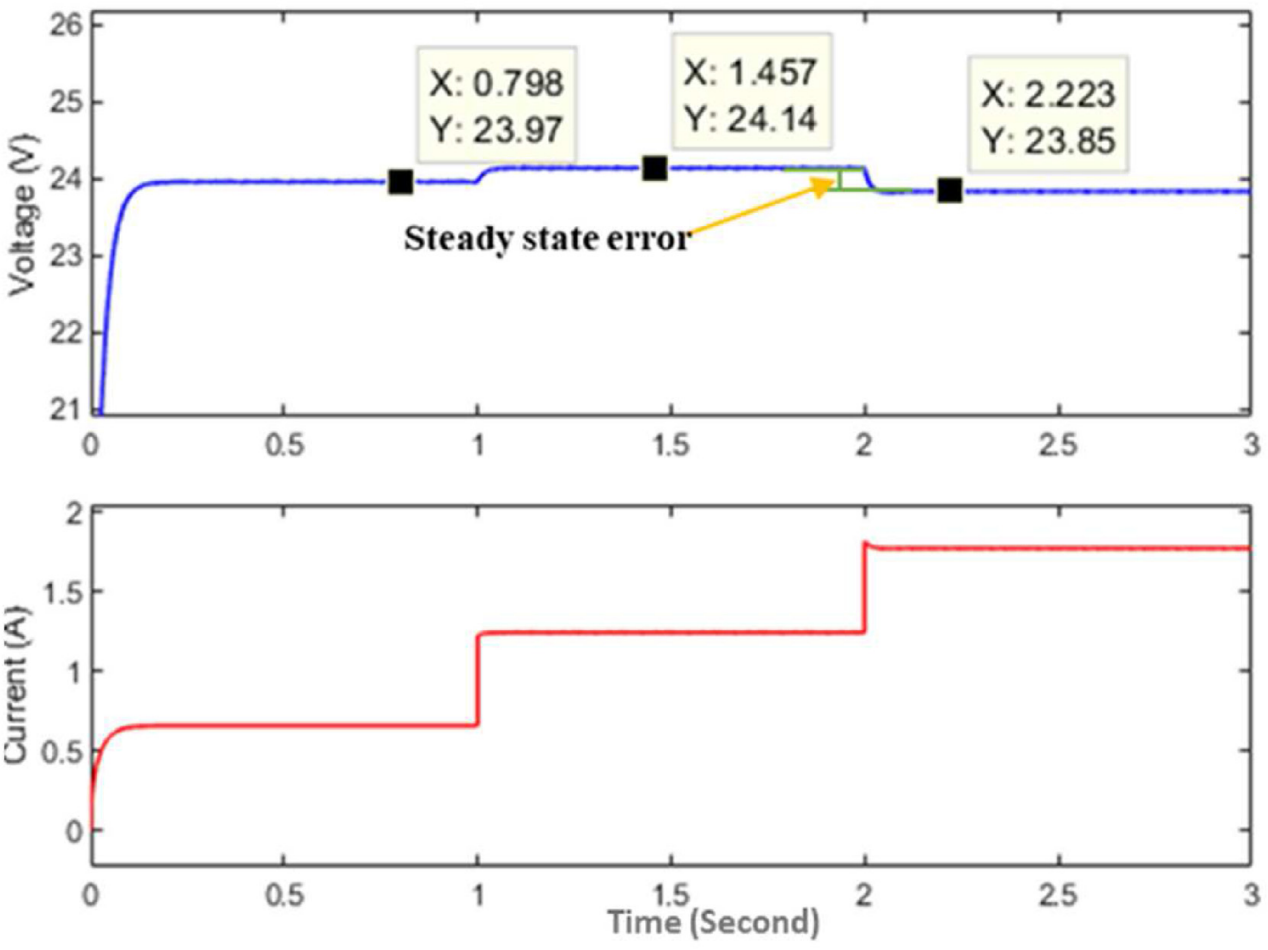
Simulation response of the converter from the non-linear model.

Where, *a* = 6.568, *b* = -0.5434, *c* = 0.3144, *d* = -0.03235. The boost converter used in this experiment is designed to give a maximum output current of 4 *A* with an inductor size of 43 *uH* and an output capacitor of 4000 *uF*. After the training, the policy is implemented without a compensator to observe the response with the policy alone as the controller. The response of the converter with the policy function as a controller is shown in Figure 7.

**Figure 8:**
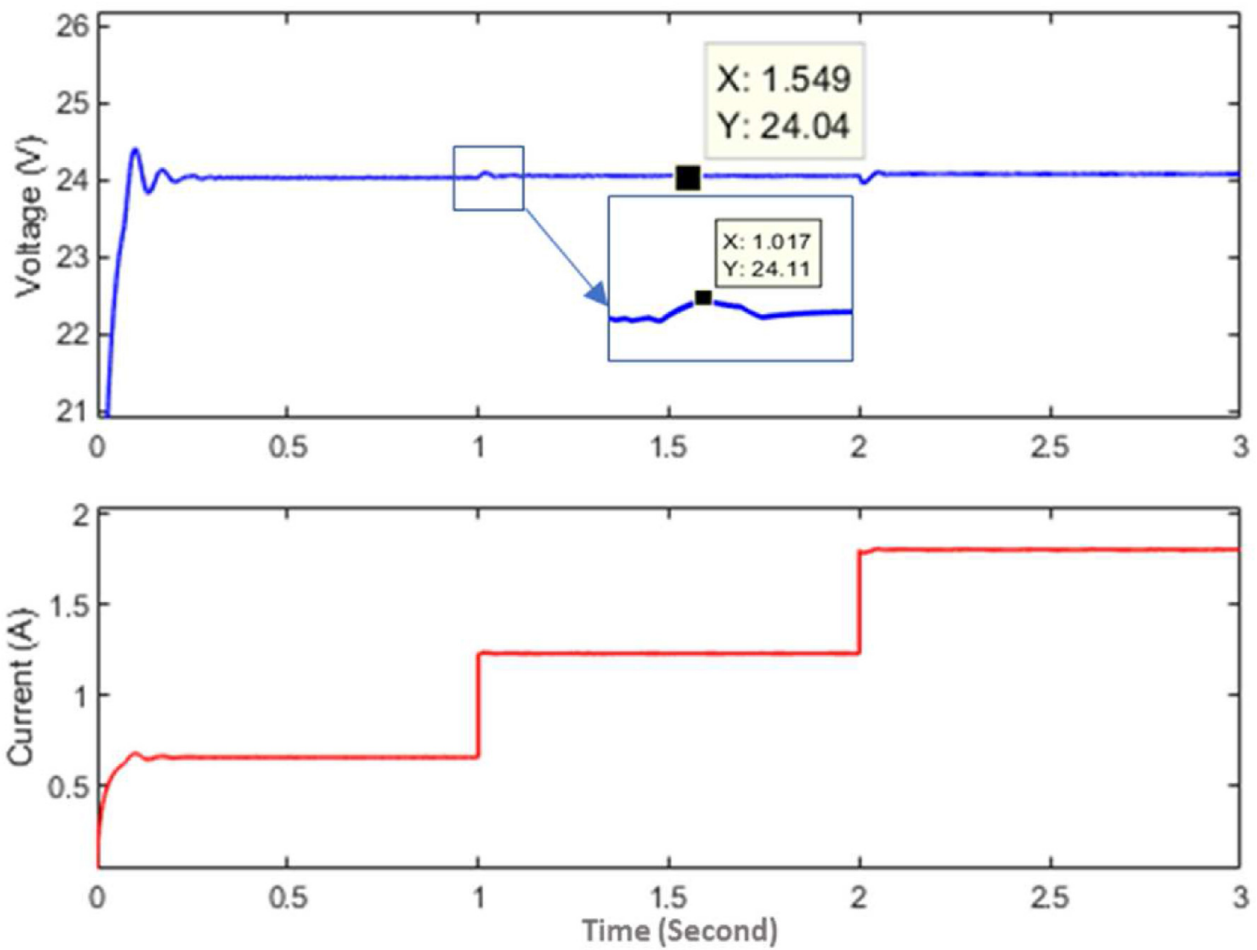
Simulation response of the proposed controller.

**Figure 9:**
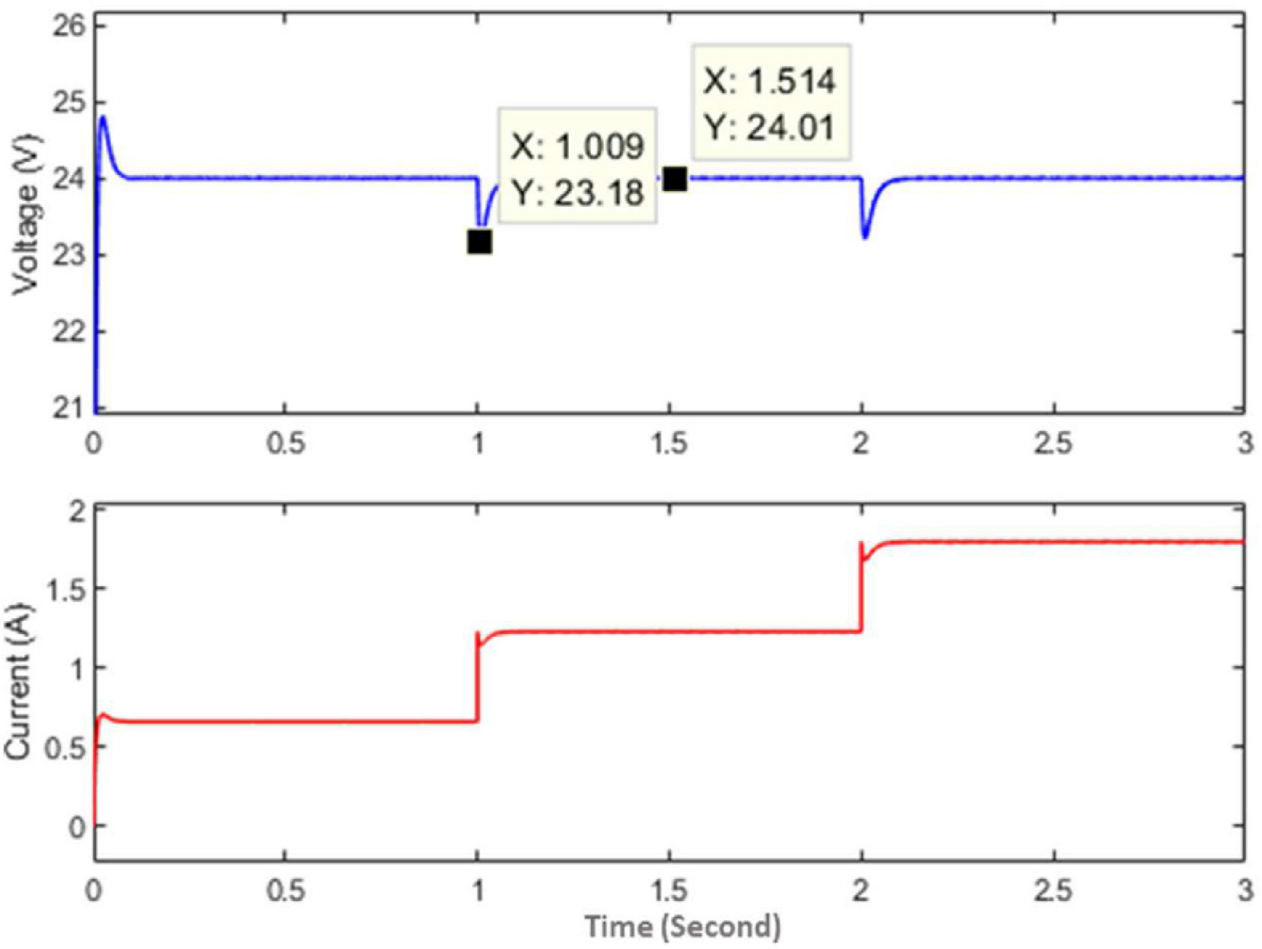
Simulation response of PI controller.

As it can be seen from Figure 7, the output has a very low transient error, however, there is a steady-state error due to imperfections during input-output mapping and a limited number of data used to determine the policy function. The policy function generates the response instantly with minimum transient voltage fluctuation, but it might generate a small steady-state error due to imperfections in mapping and approximation. This steady-state error is eliminated by using an integral compensator. After adding the compensator, the response of the controller can be seen in Figure 8. The response of the proposed controller is compared with the response of a PI controller, and the response generated by the PI controller in the same converter can be seen in Figure 9.

As can be seen from Figures 8 and 9, the proposed controller gives better transient stability than a PI controller when it is implemented to control the boost converter. Further analysis of hardware has been done to analyze the response of the controller when implemented on hardware.

### 4.2 Experimental outcomes

**Figure 10:**
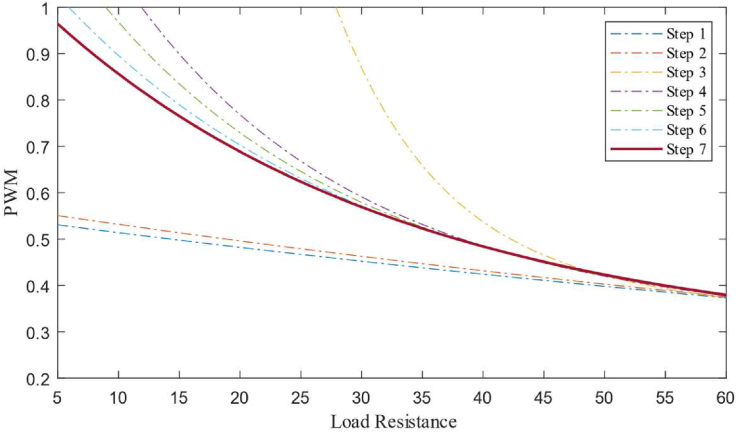
Policy function at different levels of training (Hardware).

A boost converter with an inductor of 40 *uH*, switching MOSFET operating at 10 kHz and an output capacitor of 4000 *uF* is used in this experiment. An esp32 based controller is used to run the control algorithm with a sampling time of 1 ms. Load switching is obtained by using the relays for every 5 s to train the policy function. A non-linear regression algorithm is coded on the controller to optimize the policy function based on the data gathered at the *Q* table at different stages of the training. Figure 10 shows how the policy function optimizes over different stages of training, and the final policy function derived is given by Eq. (23).

(23) $$F\left(R_{L}\right)=ae^{bR_{L}}+ce^{dR_{L}}$$

**Figure 11:**
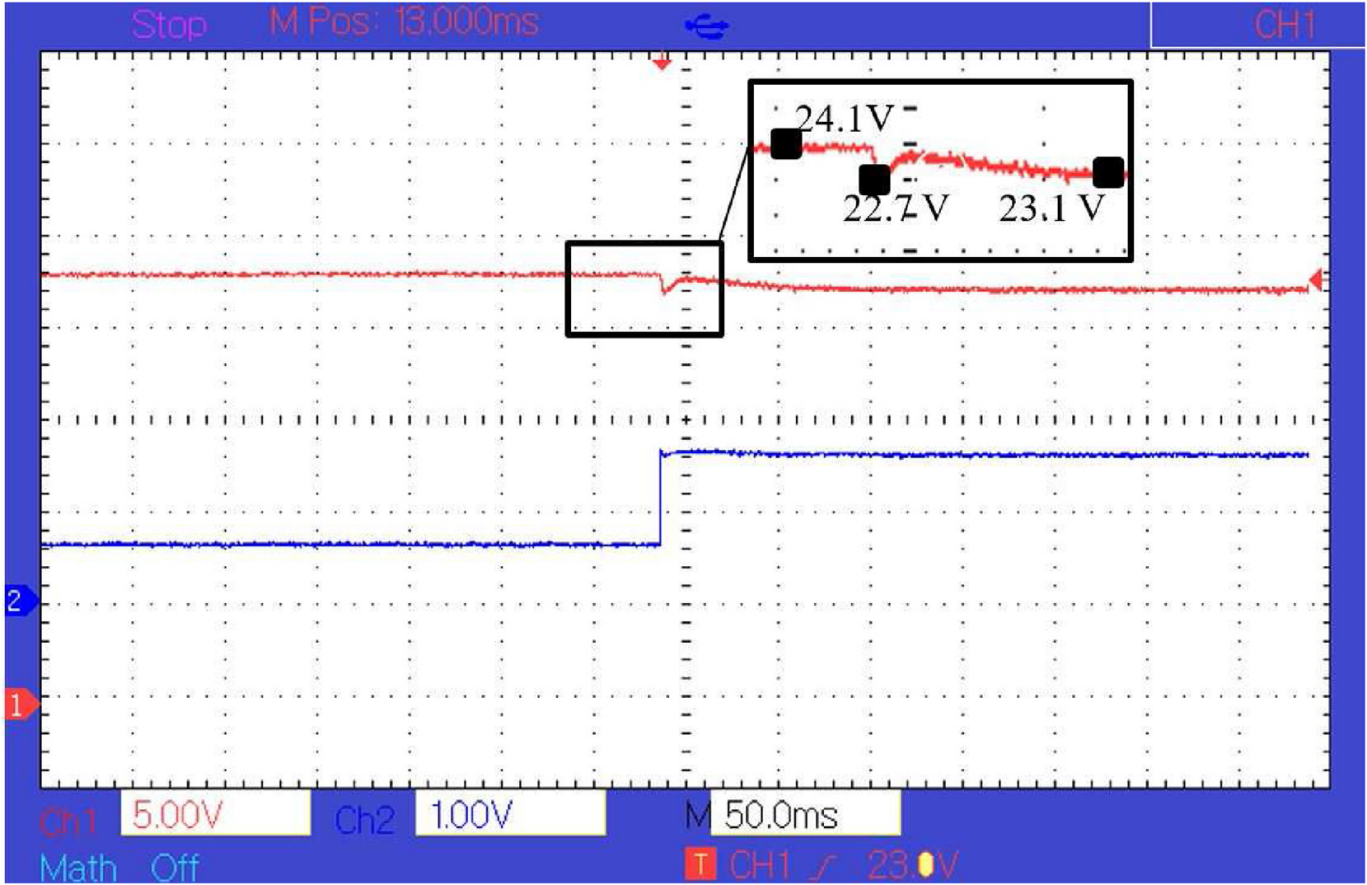
Response of the controller with policy function as a controller.

Where, *a* = 642, *b* = -0.03442, *c* = 231.4, *d* = -0.000678. Figure 10 shows the policy function at different levels of training from Step 1 to Step 8. As the training progresses, the controller switches load at the converter output, because of which, the number of state-action-pair available for policy function optimization increases so that the policy function becomes better and better with each step. The policy function is then used by the controller to generate the required PWM for a given load condition. The experimental response of the controller based on policy function alone can be seen in Figure 11.

**Figure 12:**
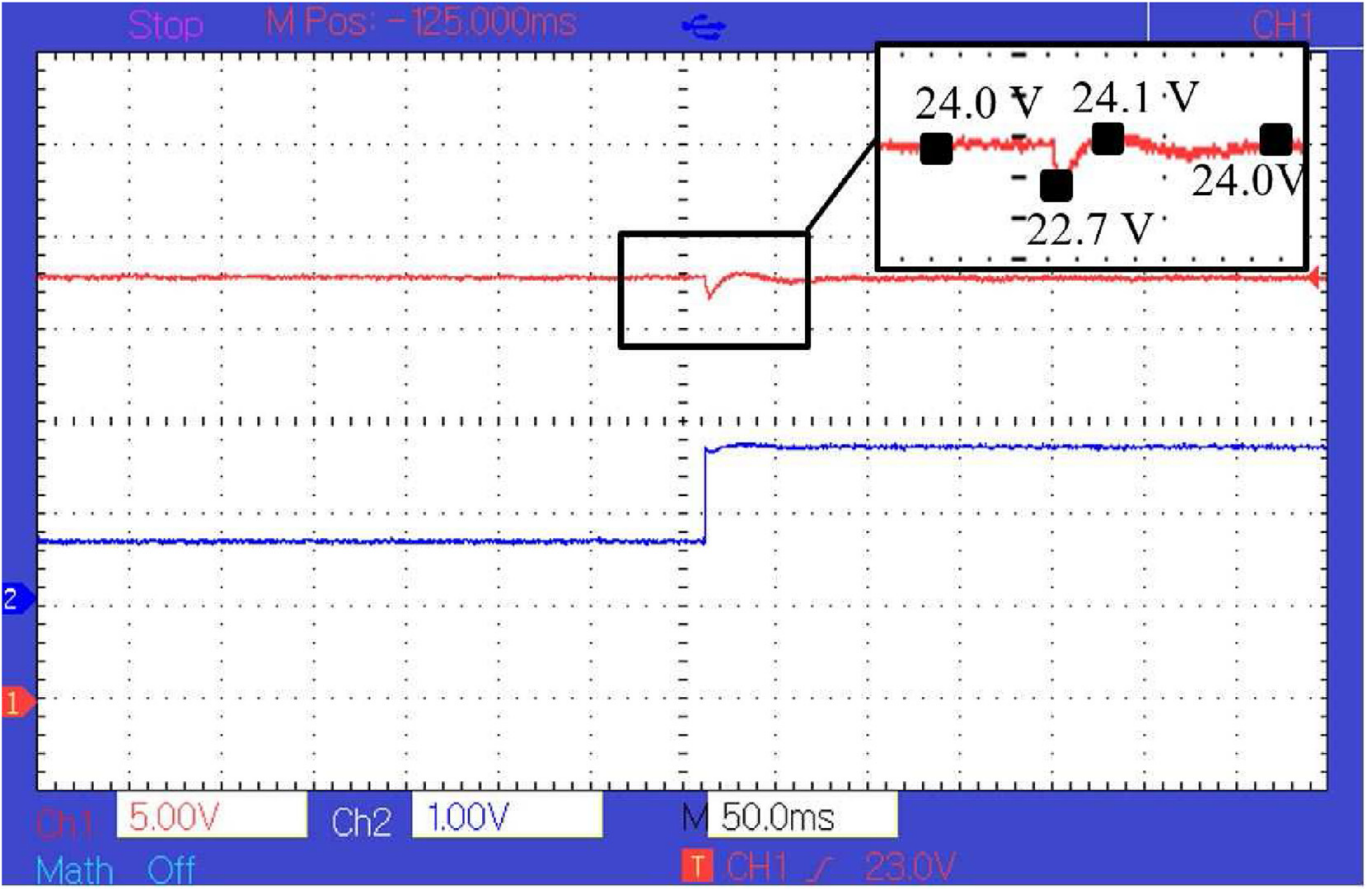
Response of the controller after policy function is paired with compensator.

**Figure 13:**
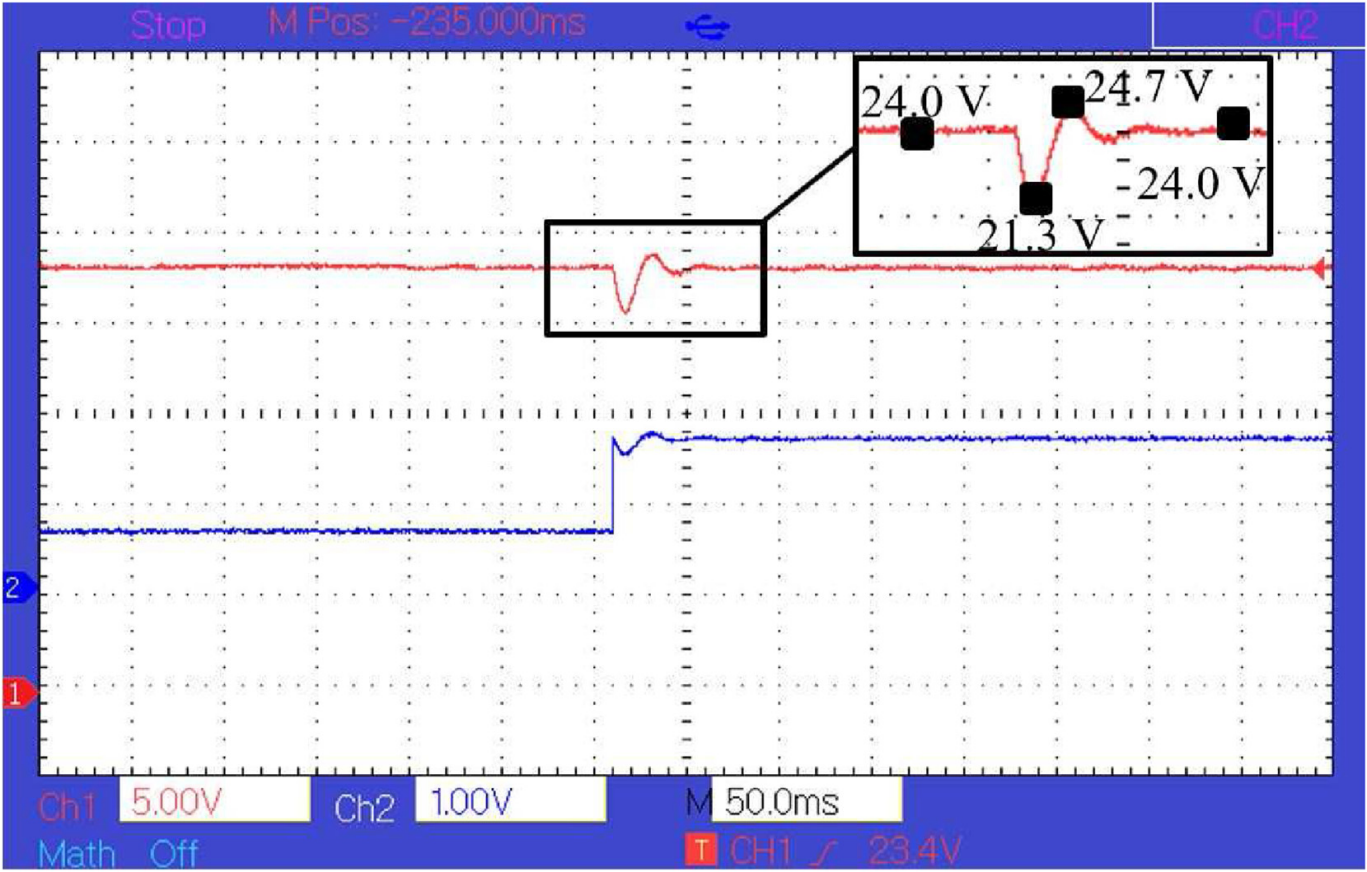
Response of the PI controller.

As can be seen in Figure 11, there is a constant steady-state error of about 0.5 *V* and transient fluctuation of about 1 *V*. The transient voltage fluctuation is less than the transient fluctuation generated by the PI controller which is about 3.5 *V* as shown in Figure 13. Although the transient voltage fluctuation is reduced compared to PI controller there is still some steady-state error that needs to be eliminated. To eliminate the error, a compensator is paired with the policy function. The response of the controller after the policy function is paired with a compensator can be seen in Figure 12. In Figures 11, 12, and 13, the voltage is measured at 5 *V* per division and the current at 1 *V* per division. The current is measured concerning the voltage drop across a shunt resistor of 0.47 *Ω*. Each division of the graph represents 2.12 *A* of current. The time scale is represented in terms of 50 ms per division, and the sampling time is 1 ms. It can be seen from Figures 12 and 13, that the peak transient voltage fluctuations of the proposed controller are about 1.5 *V* and of the PI controller is about 4 *V* similarly settling time is about 30 ms for the proposed controller and about 50 ms for PI controller. Hence, both transient fluctuations and settling time are reduced by the proposed controller at a similar sampling frequency.

It can be seen that the response signal generated by the proposed controller is faster and has less transient voltage fluctuation than the traditional PI controller. It also helps in reducing transient instability. The response of the converter controlled by the proposed model in simulation and hardware can be seen in Figures 10, 11, and 12. It is clear from Figures 8, 9, 12, and 13 that the response time and transient stability of the proposed controller are better than that of a conventional PI controller.

**Figure 14:**
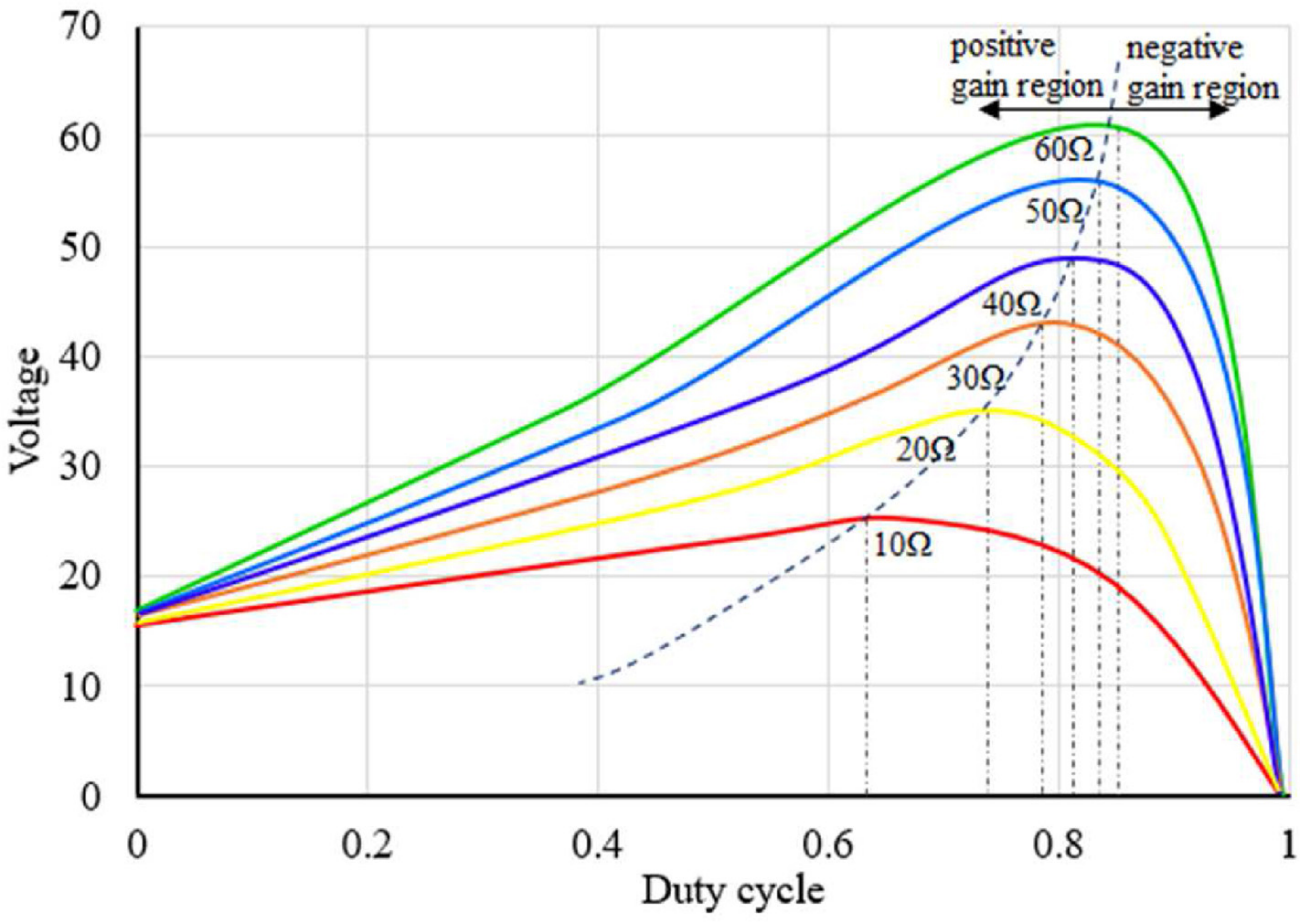
Duty cycle vs voltage for the boost converter used in this experiment.

For the dynamic duty cycle limiter, a support vector is determined to separate positive and negative gain regions. While running a converter, in order to maximize efficiency and avoid damaging the converter, the converter should be run in a positive voltage gain region. To determine the support vector separating the positive gain region and negative gain region, a non-linear regression is used in this experiment. Figure 14 shows how the positive and negative gain point is separated for different load condition for the converter used in this experiment.

(24) Duty limit = -0.001R^2^ + 0.010R + 0.545

**Figure 15:**
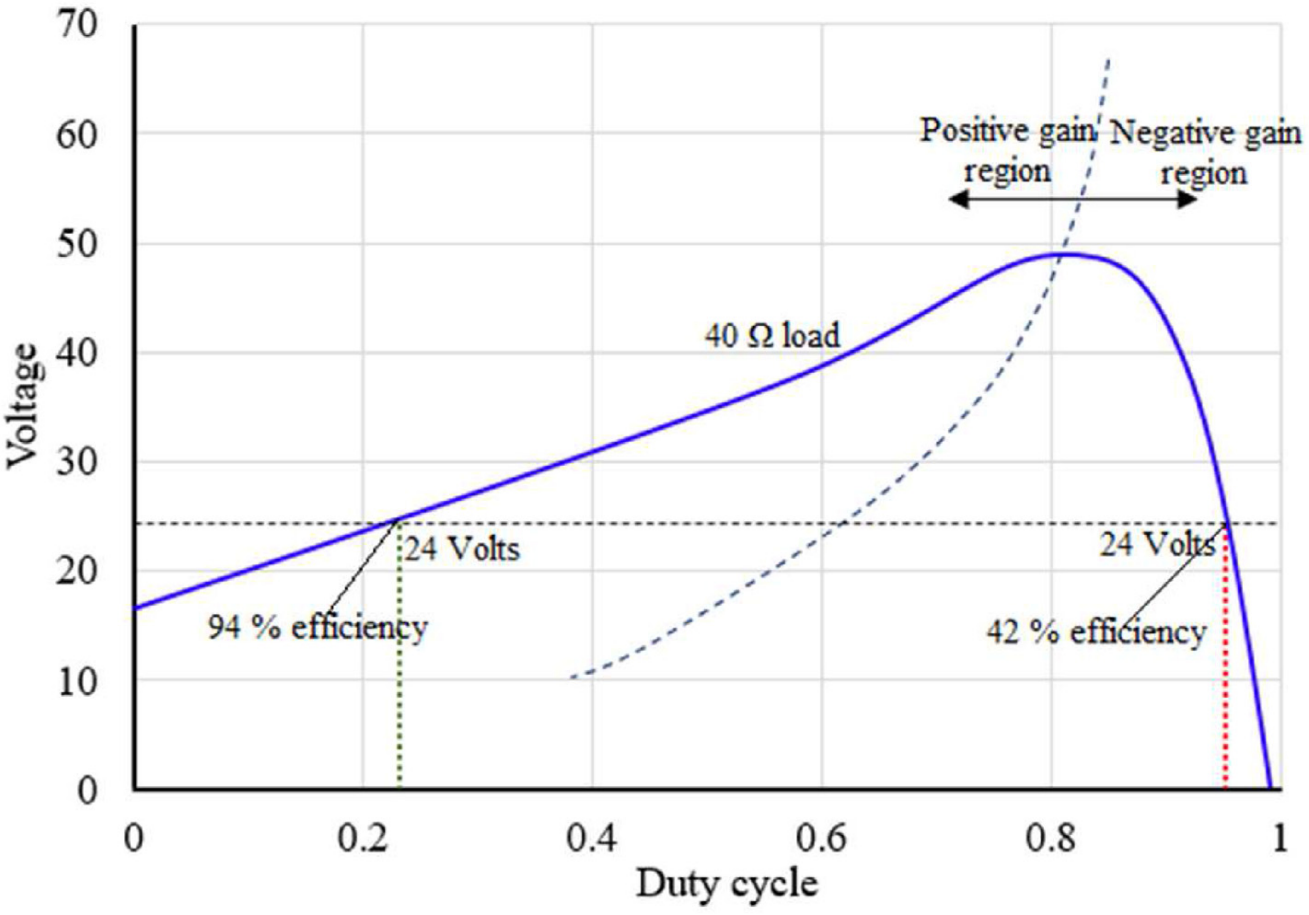
Converter running with different efficiency for the same load on positive and negative gain region.

Using a support vector to separate positive and negative gain regions and using this support vector to run the converter, helps to run the converter efficiently. Figure 15 shows a condition in which a converter can output similar voltage in both positive and negative gain regions, but with different operating efficiency, and Eq. (15) represents the second order equation separating positive and negative gain regions. The converter is maintaining 24 *V* constant output in both positive and negative gain regions, but in the positive gain region, it has much higher efficiency than that of the negative gain region. By using the support vector method combined with regression-based optimization, the controller easily avoids the negative gain region and helps run with maximum efficiency.

## 5 Conclusion

In this study, a robust controller for the DC/DC boost converter has been proposed and verified experimentally. As it can be seen from the simulation and experimental-based outcomes, the proposed controller significantly improves the transient and steady-state stability of the converter as compared to traditional controllers like PI controllers. The proposed controller combines a non-linear model and an integral controller to form a hybrid controller. The reinforcement learning model maps the non-linearity of the converter and uses the mapped model as a policy to generate control signals. The same reinforcement learning model can also be used to dynamically assign duty cycle limits depending upon the load. A dynamic duty cycle limitation method has been implemented using reinforcement learning. In future works, the proposed controller can be improved and made further robust by considering more variables like input voltage in the policy function. The proposed hybrid controller can be integrated with other DC/DC converters and inverters for voltage and frequency regulation.

## Declarations

### Author contribution statement

Anup Marahatta: Conceived and designed the experiments; Performed the experiments; Analyzed and interpreted the data; Wrote the paper.

Yaju Rajbhandari: Performed the experiments; Analyzed and interpreted the data; Wrote the paper.

Ashish Shrestha, Anup Thapa: Conceived and designed the experiments; Analyzed and interpreted the data.

Sudip Phuyal: Performed the experiments; Contributed reagents, materials, analysis tools or data.

Petr Korba: Analyzed and interpreted the data; Contributed reagents, materials, analysis tools or data.

### Funding statement

This study was supported by the EnergizeNepal Project Office, Kathmandu University, Nepal (PID: ENEPRENP-II-19-03).

### Data availability statement

Data will be made available on request.

### Declaration of interests statement

The authors declare no conflict of interest.

### Additional information

No additional information is available for this paper.
